# Annexin A5 reduces early plaque formation in ApoE -/- mice

**DOI:** 10.1371/journal.pone.0190229

**Published:** 2017-12-21

**Authors:** Robert Stöhr, Leon Schurgers, Rick van Gorp, Armand Jaminon, Nikolaus Marx, Chris Reutelingsperger

**Affiliations:** 1 Medizinische Klinik I, RWTH Aachen University, Aachen, Germany; 2 Cardiovascular Research Institute Maastricht Department of Biochemistry, University Maastricht, Maastricht, The Netherlands; Max Delbruck Centrum fur Molekulare Medizin Berlin Buch, GERMANY

## Abstract

Annexin A5 (AnxA5) exerts anti-inflammatory, anticoagulant and anti-apoptotic effects through its binding to cell surface expressed phosphatidylserine. We previously showed that AnxA5 can stabilize advanced atherosclerotic plaques by reducing macrophage infiltration. We now investigated the effects of AnxA5 administration on the onset of atherosclerosis development. Eight-week-old ApoE-/-mice were fed a western diet while being administered AnxA5 or control (M1234) for a total of 6 weeks. AnxA5 administration reduced plaque size in the aortic root as well as the aortic arch by 36% and 55% respectively. As determined by immunohistochemistry, administration of AnxA5 further stabilized plaque by reducing macrophage content and increasing smooth muscle cell content. Furthermore, the pre-treatment of HUVEC’s with AnxA5 reduced monocyte adhesion under flow-conditions. Finally, AnxA5 administration results in a trend to reduced cell death more pronounced in the aortic arch than the aortic root. In conclusion, treatment with AnxA5 before the onset of atherosclerosis reduces plaque formation in a murine model of atherosclerosis in part by reducing apoptotic rates further to its beneficial effect on macrophage infiltration and activation.

## Introduction

Atherosclerosis is an inflammatory process driven by the accumulation of cholesterol rich particles in the vascular endothelium[1, 2]. This process leads to a process of „endothelial dysfunction“, which in turn initiates the recruitment of circulating monocytes to the site of injury. Once inside the plaque, monocytes differentiate to active macrophages and start ingesting the cholesterol particles mainly composed of oxidized low density lipoprotein (LDL) and the release of pro-inflammatory cytokines to further attract monocytes[3]. Overwhelming ingestion of LDL leads to cellular death of macrophages resulting in the exposure of apoptotic material which potentiates inflammation with more monocyte recruitment. This accumulation of immune cells, mainly driven by continuous low grade inflammation leads to growth of the plaque with the potential for occlusion of the affected vessel[4].

Since the main initiation for the development of atherosclerosis is the accumulation of LDL the mainstay of prevention involves the reduction of circulating LDL[5]. Once a plaque has been established however, there is also need to reduce the ongoing inflammation to promote plaque stability. As such statins have proven an invaluable tool thanks to their pleiotropic effects which lead to a reduction in plaque inflammation on top of their influence on cholesterol metabolism[6]. However, many patients taking statins still develop complications of atherosclerosis and thus further tools are needed to reduce plaque buildup. Reducing inflammation in the early, just established plaque is a very interesting target which has shown great promise in animal models. As such, preventing the exposure of apoptotic material inside the plaque may be able to reduce macrophage influx and thus plaque growth[7].

Annexin A5 (AnxA5) is a single chain protein that belongs to the annexin gene superfamily and is known for its high affinity binding to phosphatidylserine (PS) in a calcium-dependent manner. PS is considered a DAMP that becomes accessible on the surface of stressed and dying cells and extracellular vesicles (EVs). *In vitro* studies have shown that AnxA5 binds to cells and EVs expressing PS and, consequently, suppresses their PS-mediated pro-inflammatory actions[8]. *In vivo* AnxA5 dampens inflammation when administered to various mouse models [9–11]. Recent evidence also points to AnxA5 as a modulator of T-cell activation by reducing LDL mediated expression of the Human Heat Shock Protein 60 (HSP-60) [12].

Our group has demonstrated that AnxA5 reduces inflammation in advanced atherosclerosis and contributes to stabilization of the advanced plaques in the ApoE-/- mouse model of atherosclerosis [13]. This paper addresses the therapeutic potential of AnxA5 during the early phase of atherogenesis in the ApoE-/- mouse model. We demonstrate that administered AnxA5 reduces inflammation and suppresses early plaque development in a PS-dependent manner.

## Materials and methods

All animal experiments were approved and carried out in strict compliance with the University of Maastricht Institutional Animal Care and Use Committee (IACUC) guidelines and in accordance with the “Guide for the Care and Use of Laboratory Animals” (1996) by the Institute of Laboratory Animal Research Commission on Life Sciences (ILARCLS, National Research Council, Washington, D.C.)

### Expression and purification of AnxA5

AnxA5 and M1234 were prepared as described previously[14]. Briefly, AnxA5 and M1234 were expressed in *Escherichia coli* M15 (*pREP4*) (Qiagen, Venlo, the Netherlands), which were transformed with pQE30Xa (Qiagen) containing cDNA of human AnxA5 and M1234. Bacteria were harvested and lysed by sonification. Cell debris was removed by centrifugation. His-tagged proteins were isolated from supernatant by chromatography using nickel columns (GE Healthcare, Eindhoven, the Netherlands) and an imidazole gradient. Purified proteins were checked on homogeneity (MALDI-TOF/TOF) and PS binding activity (ellipsometry) as described elsewhere. All recombinant protein samples contained less than one endotoxin unit per ml as determined by Endosafe PTS spectrophotometer (Charles River, Leiden, the Netherlands).

### ApoE^−/−^-mouse model of atherosclerosis

Atherosclerotic lesions were induced in 8 weeks-old male ApoE−/− mice using both a Western type diet (0.25% cholesterol) (Abdiets, Woerden, the Netherlands). After one week, AnxA5 (1 mg/kg) or M1234 (1 mg/kg) were injected i.p. three times a week. The dosage and route of application used in our mouse experiment is similar to what has been described to be effective in murine models by other groups and is based on a blood clearance study in which we determined the half-life of AnxA5 following i.p. injection of radiolabelled AnxA5 to be 5.64 ± 2.33 hrs. Mice were killed by isoflurane overdose after another 6 weeks of Western type diet feeding and the aortic arch as well as the aortic root were excised for immunohistochemical analysis. Blood was collected for analysis of leucocyte subsets using flow cytometry.

### Histology and immunohistochemistry

Paraffin sections were stained with haematoxylin/eosin (Merck/ Roth) to determine the volume of atherosclerotic lesions. Quantitative analysis of lesions was performed with Image J software. Parallel sections were stained with monoclonal rat anti-mouse Mac2 (Cederlane), TUNEL (*in situ* cell death detection kit, POD—Roche Applied Science, Woerden, the Netherlands) and αSMactin (Dako) to stain macrophages, apoptotic cells and smooth muscle cells, respectively.

Gomorri staining was performed to analyze the amount of collagen as described elsewhere.

### Flow cytometry

Using subset-specific antibodies (all purchased from BD Bioscience) we analyzed T cells, B cells, NK cells, granulocytes, monocytes and relevant subsets of these populations as described elsewhere. All measurements were performed on a FACS Canto II (BD Biosciences, Breda, the Netherlands) and analysis of acquired data was performed with FACS Diva software (BD Biosciences).

### Flow chamber assay with human monocytes and activated HUVEC cells

Human umbilical vein endothelial cells (HUVEC) were seeded on gelatin-coated Slides VI0.4 (Ibidi, Germany) and confluent monolayers were stimulated with TNF-α(10ng/ml; R&D Systems, United Kingdom) overnight. Human peripheral blood mononuclear cells (PBMCs) were freshly isolated from healthy volunteers by double- density gradient centrifugation. PBMCs were diluted to 1*106/mL in Dulbecco phosphate buffered saline (DPBS) supplemented with Ca2+ and Mg2+, and incubated with or without AnxA5 (20 nM) at 37°C 10 minutes before the start of the flow experiment. PBMCs were perfused over the endothelial monolayers at a constant shear stress of 1 dyne/cm^2^ using a syringe pump (Harvard Apparatus, South Natick, MA). After 8 minutes of perfusion, 6 random fields were recorded for 10 seconds ready for off-line analysis. Video sequences were transferred to a computer and loaded into ImageJ software. PBMCs were manually tagged and their movements on the endothelium monitored. Adherent monocytes were quantified and shown as percentage of captured monocytes in the vehicle group.

### Statistics

Data are presented as means ± SEM. A two-tailed Student’s t-test was used to determine significance unless stated otherwise. Differences were considered significant for p<0.05 (*) and p<0.01 (**)

## Results

### AnxA5 administration does not affect mouse characteristics and circulating immune cells

Repeated injections of AnxA5 do not affect development of mice. There was no mortality recorded during the experimental period in either group (Table 1). Weight development was similar between the 2 groups as were cholesterol levels and circulating leucocyte subsets as analyzed by flow cytometry (Table 1).

**Table 1:** Animal data.

	anxA5 (n = 8)	M1234 (n = 8)	P
**Weight (gr)**	**29,30 ± 1,023**	**28,40 ± 0,7775**	**ns**
**Mortality**	**0**	**0**	**ns**
**Cholesterol mg/dl**	**756,6 ± 33,29**	**690,8 ± 16,70**	**ns**
**FACS analysis**			
**B cells (% live lymphocytes)**	**59,25 ± 1,333**	**62,48 ± 1,642**	**ns**
**CD3+ (%live lymphocytes)**	**15,85 ± 1,399**	**17,32 ± 1,348**	**ns**
**CD4+ (%CD3+ cells)**	**42,20 ± 5,106**	**46,84 ± 1,644**	**ns**
**CD8+ (%CD3+ cells)**	**44,50 ± 1,608**	**40,10 ± 1,768**	**ns**
**Monocytes (% non B cells)**	**45,88 ± 3,541**	**42,06 ± 2,601**	**ns**
**Ly6C-hi-monocytes (% monocytes)**	**42,20 ± 4,673**	**50,60 ± 2,746**	**ns**
**Ly6C-lo monocytes (% monocytes)**	**32,30 ± 8,423**	**29,14 ± 2,277**	**ns**

### AnxA5 reduces early atherosclerosis development

**Fig 1:**
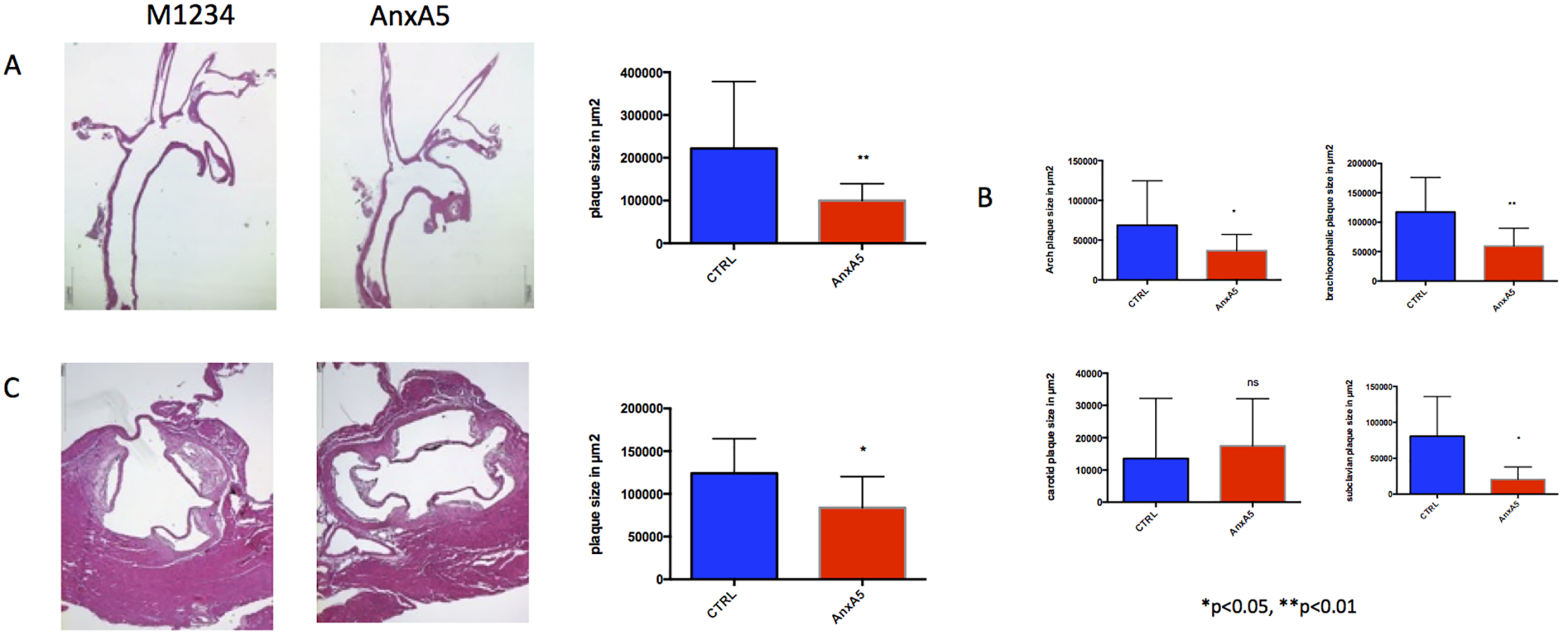
Administration of AnxA5 reduces atherosclerotic development in ApoE-/- mice. Representative H&E images of the A) aortic arch, B) its branches as well as C) the aortic root. *p<0.05 ** p<0.01, n = 16–18.

After 6 weeks of western diet, ApoE -/- mice treated with AnxA5 revealed a reduction in atherosclerosis development in the aortic root (130027 ± 16988 vs. 83766 ± 9408 **μm**^**2**^ p = 0.0242) as well as the aortic arch (221811 ± 39118 vs. 99770 ± 9922**μm**^**2**^ p = 0,0077) when compared to animals treated with the control compound M1234 (Fig 1A). Plaque development was consistently reduced in all vessels included in the aortic arch, excluding the carotid artery (Fig 1B) suggesting a generalized mechanism, rather than a flow associated effect.

### Administration of AnxA5 reduces plaque apoptotic burden

**Fig 2:**
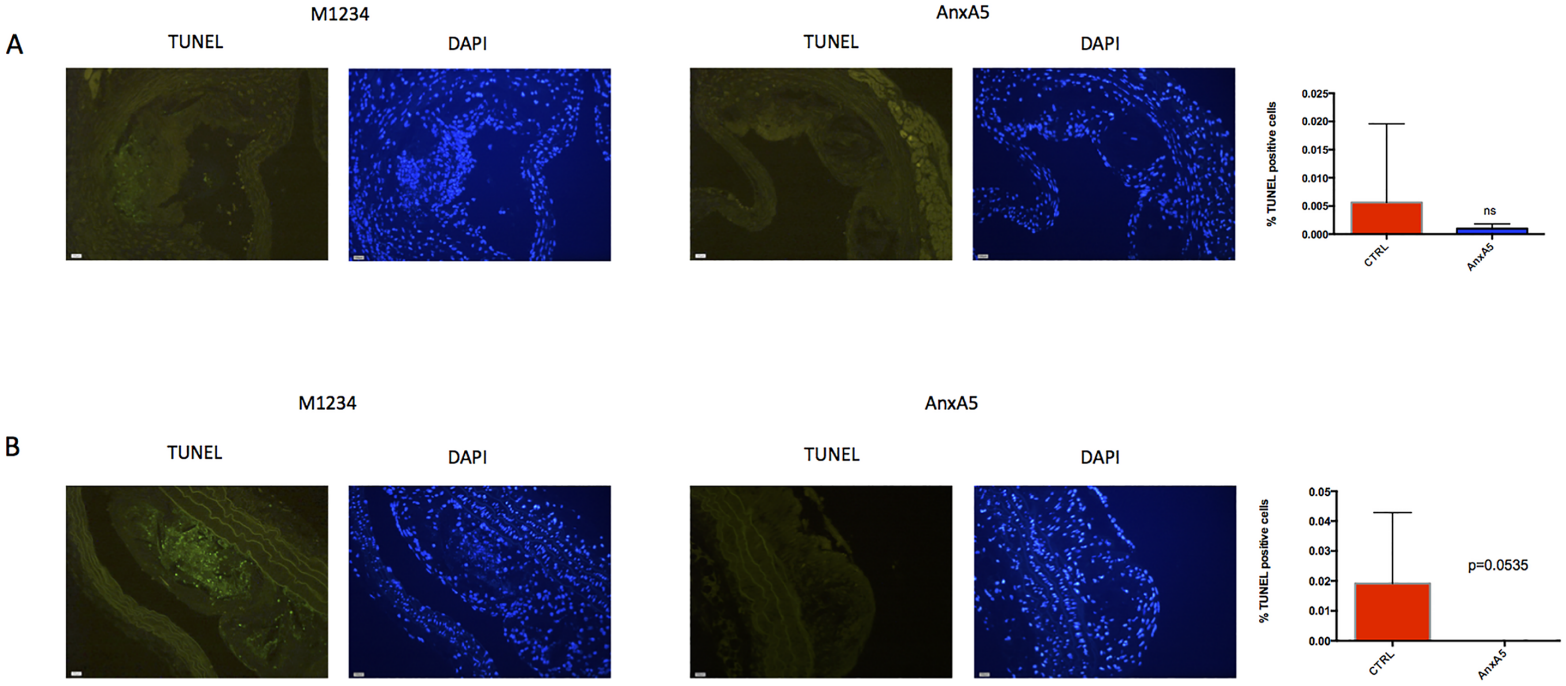
AnxA5 reduces cell death. Representative images of TUNEL stains from A) the aortic root and B) the aortic arch. n = 6 per group.

Since AnxA5 has been proven to reduce apoptosis in vitro[15], we performed TUNEL analysis to evaluate the apoptosis rate. Whereas in the aortic root the reduction was not significant (Fig 2A), we did find a strong trend to a reduction of apoptosis in the aortic arches (Fig 2B). Furthermore, in the aortic arches, there was no evidence of any apoptosis at all.

### AnxA5 but not M1234 administration reduces macrophage infiltration and increases plaque stability

**Fig 3:**
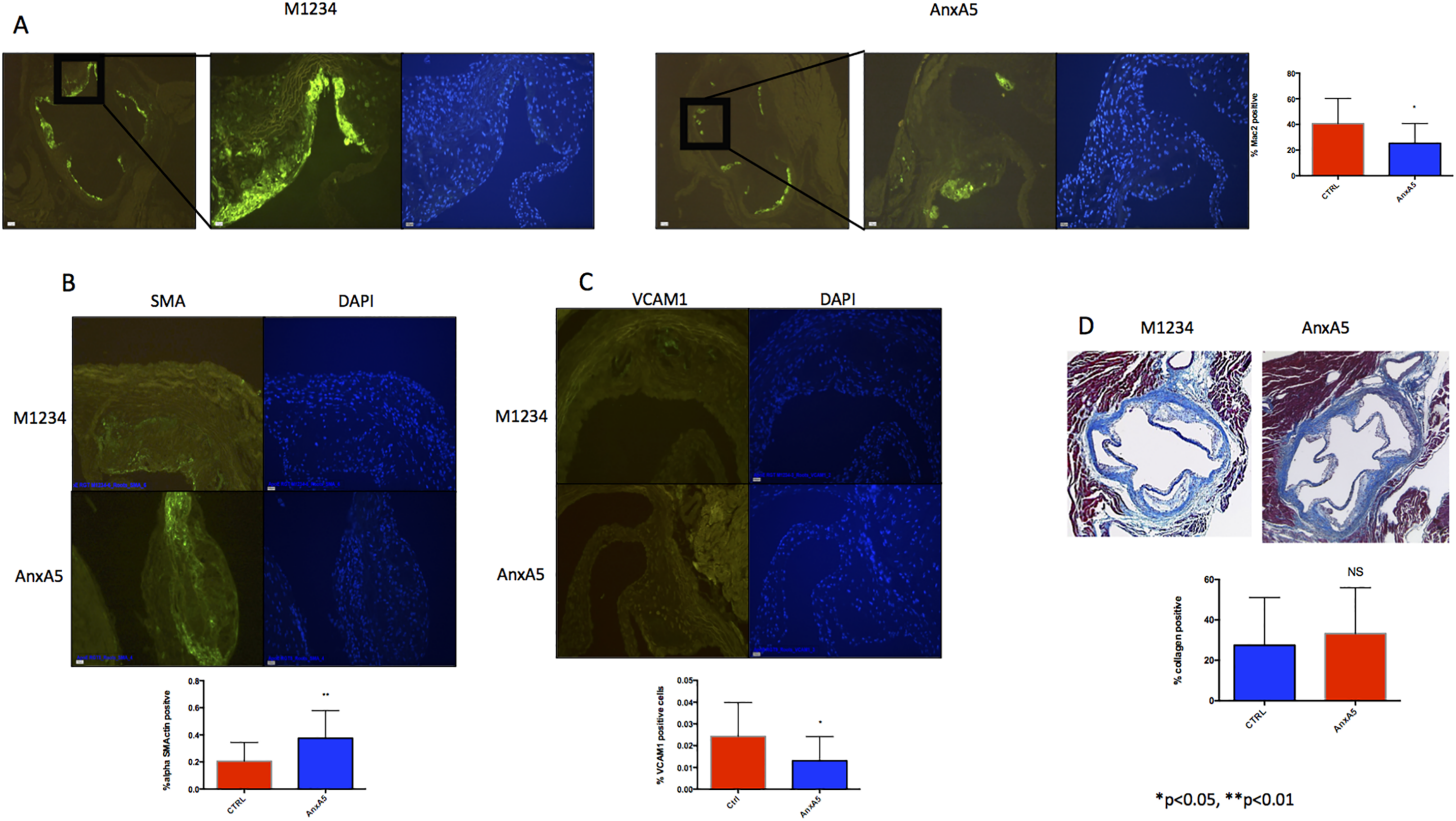
AnxA5 administration reduces macrophage infiltration and stabilizes plaque development. Representative images of A) Macrophage infiltration (Mac-2) B) Smooth muscle cell content (alpha-Smooth Muscle Actin) C) VCAM1expression and D) plaque collagen content (Gomorri) *p<0.05, n = 16–18.

To evaluate plaque composition, we next performed immunohistochemistry to measure macrophage infiltration and activation by staining with Anti-Galectin 3 (Mac2). We found that administration of AnxA5 significantly reduces macrophage infiltration when compared with control in the aortic roots (25,30 ± 3,982 vs. 40,57 ± 5,254 p = 0.0272)(Fig 3A). Furthermore, the plaque of animals treated with AnxA5 showed signs of increased stability compared to the control group as demonstrated by the increased expression of alpha Smooth Muscle Actin (alpha-SMA) (0,3756 ± 0,05087 vs 0,2056 ± 0,03458 p = 0.0097) and a reduction of Vascular cell adhesion protein 1 (Vcam-1) (0,01308 ± 0,003077 vs 0,02429 ± 0,004156 p = 0.0422) (Fig 3B and 3C). There was no difference in the amount of collagen per plaque as assessed by Gomorri Trichrome staining (33,23 ± 5,677 vs 27,48 ± 5,899 p = 0.4874)(Fig 3D).

### Incubation with AnxA5 inhibits monocyte adhesion to activated endothelial cells

**Fig 4:**
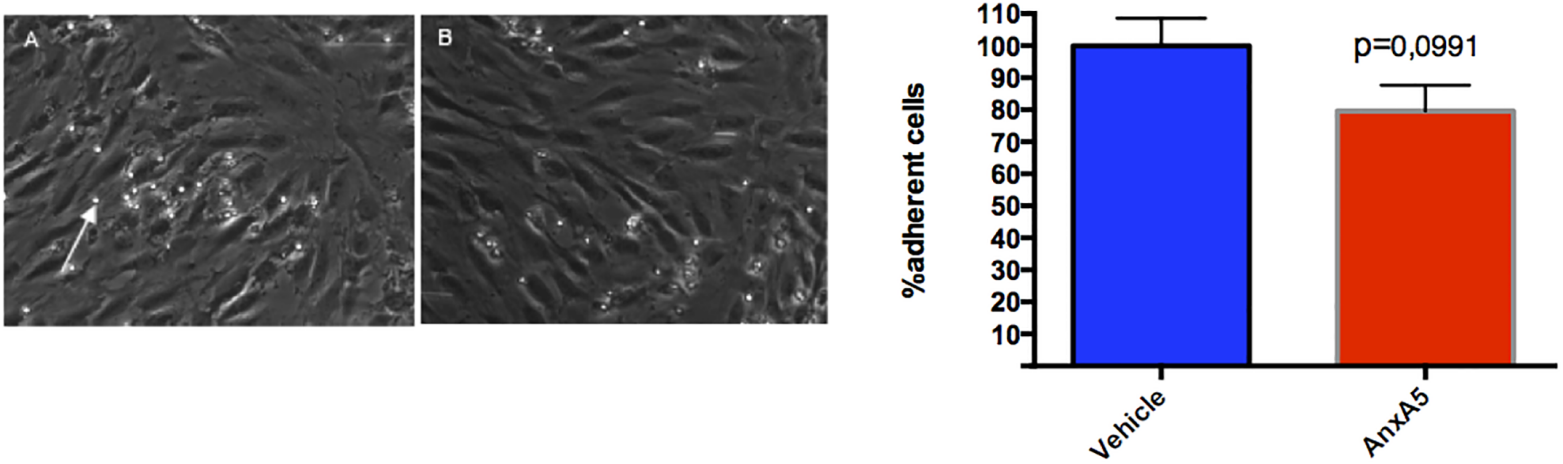
AnxA5 results in a tendency to decrease the monocyte endothelium interaction in a flow model. Representative images following the treatment of HUVEC with vehicle (A) and AnxA5 (B) prior for flow over (N = 10–12). The white arrow indicates a captured monocyte.

To evaluate the effect of AnxA5 on monocyte adhesion in vitro we performed flow-chamber experiments. To this effect activated HUVEC’s were pre-incubated with AnxA5 prior to flow over. This led to a trend for a 20% decrease in monocyte adhesion (100,0 ± 8,550 vs 79,61 ± 8,064 p = 0,0991) suggesting that inhibition of monocyte infiltration can be an additional mechanism for reduced atherosclerotic lesion formation in animals treated with AnxA5 (Fig 4).

## Discussion

AnxA5 is an endogenous protein with a high affinity for binding PS. This ability allows its usage as a marker for cellular stress and apoptosis. Furthermore, shielding of PS can, in vitro, reduce the ability of inflammatory cells to bind to apoptotic cells and thus allow cellular repair.

Therapeutic activity of AnxA5 has been shown in various mouse models of vascular inflammation [9, 10]. AnxA5 administration results in decreased recruitment of leukocytes to sites of injury, likely by acting locally in the inflamed vascular wall. The underlying mechanisms were not elucidated but hypothesized to involve binding of AnxA5 to cells exposing PS on their surface [9].

Herein, we demonstrate that PS binding of AnxA5 is mandatory to its anti-inflammatory activity in the early atherosclerotic plaque *in vivo*. We used a variant of AnxA5 (M1234) that has lost ability to bind PS at physiological calcium concentrations through one amino acid replacement in each of its 4 calcium-binding sites [16]. M1234 failed to bind PS-expressing tumor cells in a mouse model of cancer[14] and to inhibit platelet-dependent coagulation in mouse model of thrombosis [17]. Considering the accumulation of AnxA5 in advanced atherosclerotic lesions [18] we assume that PS binding of AnxA5 is also key to its ability to reduce inflammation and to stabilize advanced plaques[13]. *In vitro*, AnxA5 modulates a variety of pro-inflammatory reactions occurring at PS expressing membranes[8]. Which PS-dependent reactions are targeted by AnxA5 in atherosclerotic plaques *in vivo* have not been identified so far.

This study shows, that the administration of AnxA5 in the setting of early, onset atherosclerosis is protective and reduces atherosclerotic development as well as stabilizing the plaque. Indeed, animals treated with AnxA5 show smaller plaques in the aortic root as well as the aortic arch. The developed plaques have less infiltrated macrophages and higher expression of markers associated with plaque stability. Indeed, our flow chamber results indicate that the administration of AnxA5 can interfere with monocyte recruitment into the cell, a major process for the initiation of atherosclerosis[19].

Furthermore, we find that when administered early in the disease process AnxA5 can reduce the rate of apoptosis inside the plaque. Our group has previously shown, that in the later stages of atherosclerosis there is no difference in the apoptotic rate in the plaque.

We therefore hypothesize, that the anti-apoptotic effect of AnxA5 may be overwhelmed in the later stages of atherosclerosis due to the much higher rates in larger more complex plaques. The protective effect of AnxA5 may thus be twofold. In the earlier stages, it reduces apoptotic rates in the plaque, thus reducing macrophage infiltration and plaque growth. This effect is reinforced by the inhibition of moncoyte adhesion to the endothelial layer. In the later stages of atherosclerosis, this effect becomes overwhelmed but the anti-inflammatory effect prevails stabilizing the established plaque.

In conclusion, the treatment with AnxA5 in the early stages of atherosclerosis reduces plaque formation in a murine model of atherosclerosis in part by reducing apoptotic rates further to its beneficial effect on macrophage infiltration and activation.
